# Supplementary material for: Progressive release of long-stored carbon from tropical peatland disturbances
Source: Nat Commun. 2026 May 27;17:4369. doi: 10.1038/s41467-026-72890-y (PMC13216611; doi:10.1038/s41467-026-72890-y)
Supplement: Supplementary file 1 — Supplementary Information [file 41467_2026_72890_MOESM1_ESM.pdf]

Supplementary Information for

# Progressive release of long-stored carbon from tropical peatland disturbances

Jun Koarashi[1*], Masayuki Itoh[2], Mariko Atarashi-Andoh[1], Yoko Saito-Kokubu[3,4], Makoto Matsueda[5], Kitso Kusin[6,7], Adi Jaya[6,7], Salampak Dohong[7] and Takashi Hirano[8]

[1]Nucelar Science and Engineering Center, Japan Atomic Energy Agency, Ibaraki, Japan
[2]Research Institute for Sustainable Humanosphere, Kyoto University, Uji, Japan
[3]Tono Geoscience Center, Japan Atomic Energy Agency, Toki, Japan
[4]Integrated Support Center for Nuclear Nonproliferation, Security and Human Resource Development, Japan Atomic Energy Agency, Ibaraki, Japan
[5]Collaborative Laboratories for Advanced Decommissioning Science, Japan Atomic Energy Agency, Fukushima, Japan
[6]Center for International Cooperation in Sustainable Management of Tropical Peatland, University of Palangka Raya, Palangka Raya, Indonesia
[7]Department of Agriculture, University of Palangka Raya, Palangka Raya, Indonesia
[8]Research Faculty of Agriculture, Hokkaido University, Sapporo, Japan

*Corresponding author.
Jun Koarashi, koarashi.jun@jaea.go.jp

**This file includes:**

Supplementary Figures 1 to 6
Supplementary Tables 1 to 7
Supplementary Methods 1 to 4
Supplementary References

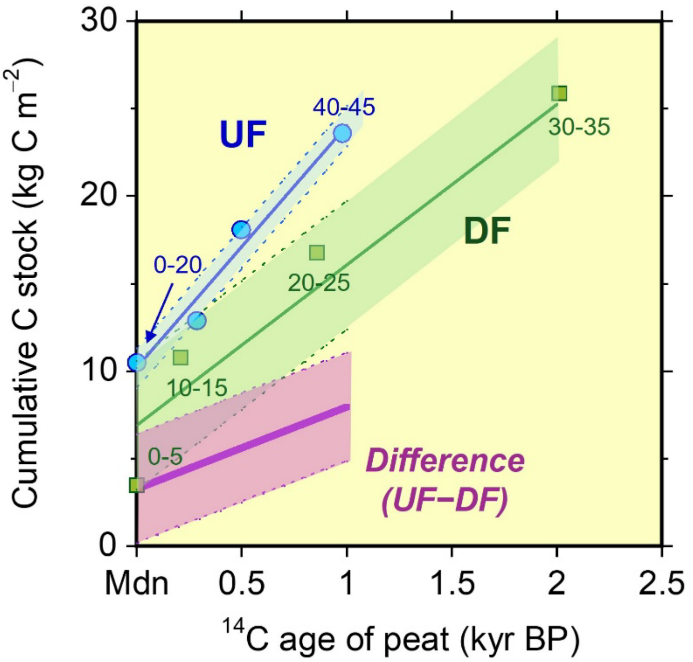


**Supplementary Fig. 1.** Comparison of cumulative C stocks versus $^{14}$C age for the UF (undrained peat swamp forest) and DF (drained forest) sites. Mdn on the X-axis denotes the modern $^{14}$C age (set to 0 kyr BP for calculations). Linear relationships shown as lines (UF: $r = 0.99$, $p < 0.05$, DF: $r = 0.99$, $p < 0.0005$) were used to estimate the difference in cumulative C stock between the sites. The estimated difference, representing drainage-induced C loss, is shown as the blue shading.

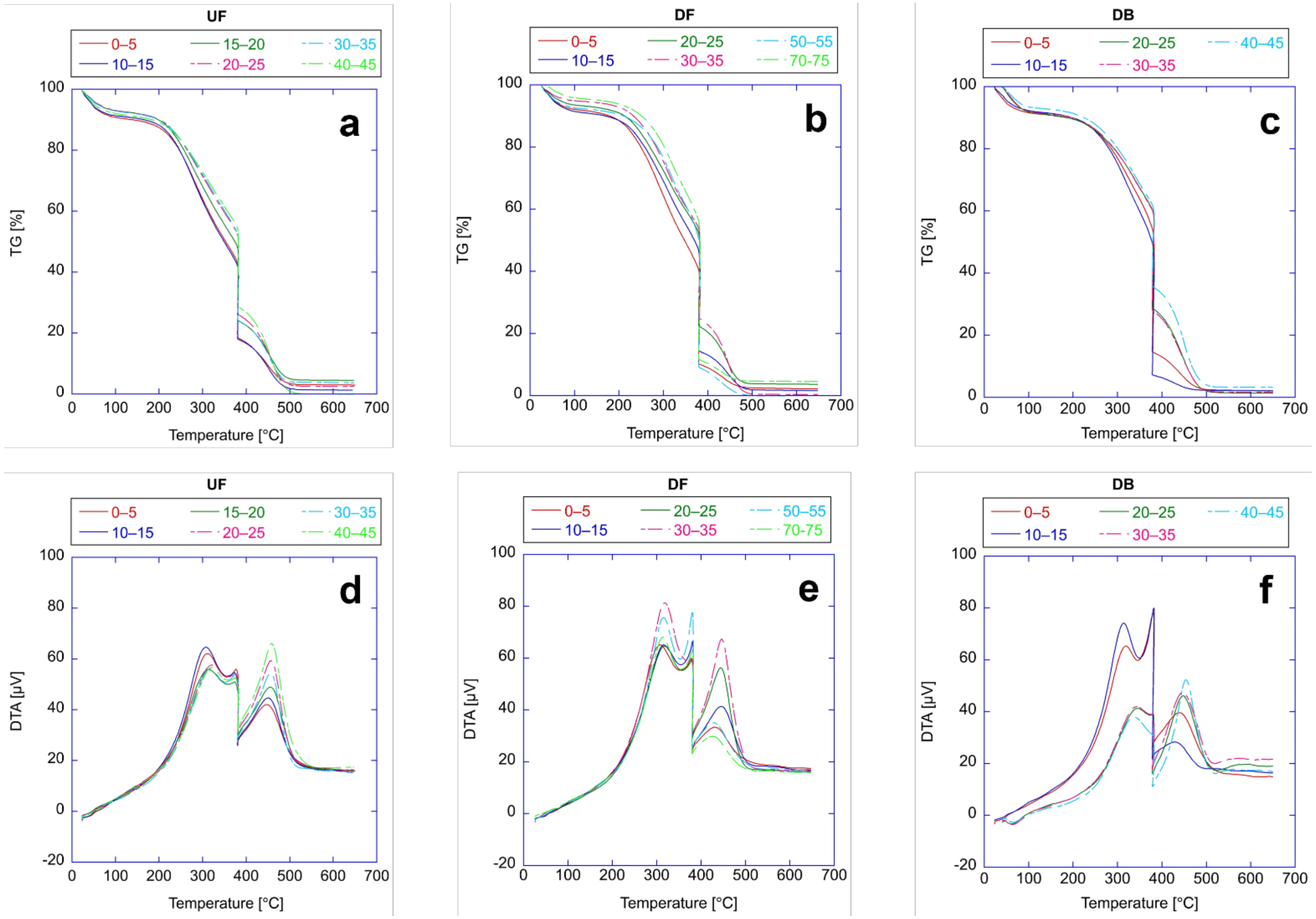


**Supplementary Fig. 2.** Thermogravimetry (TG: **a–c**) and differential thermal analysis (DTA: **d–f**) curves for peat samples from three peatland sites. UF: undrained swamp forest (**a, d**), DF: drained forest (**b, e**), and DB: drained, burnt ex-forest (**c, f**).

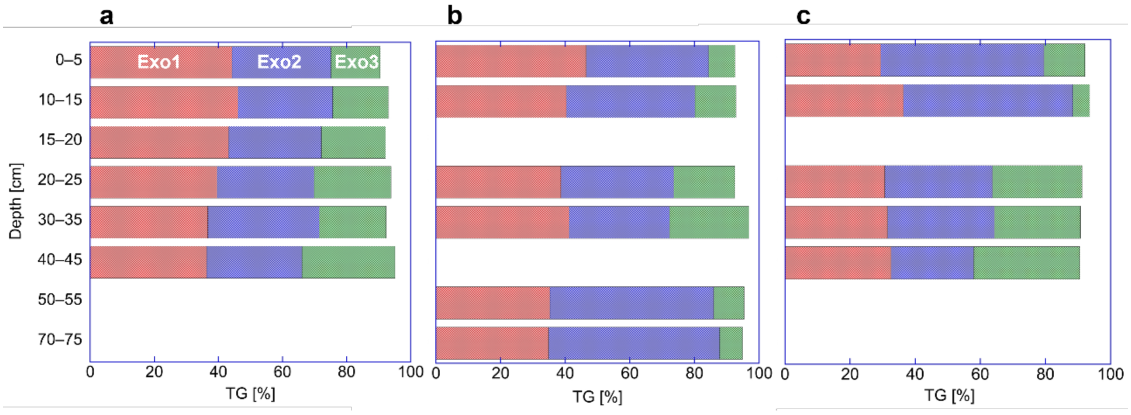


**Supplementary Fig. 3.** Relative weight losses associated with the exothermic phases (Exo-1, Exo-2, and Exo-3) in TG–DTA for peat samples from three peatland sites. UF: undrained swamp forest (**a**), DF: drained forest (**b**), and DB: drained, burnt ex-forest (**c**).

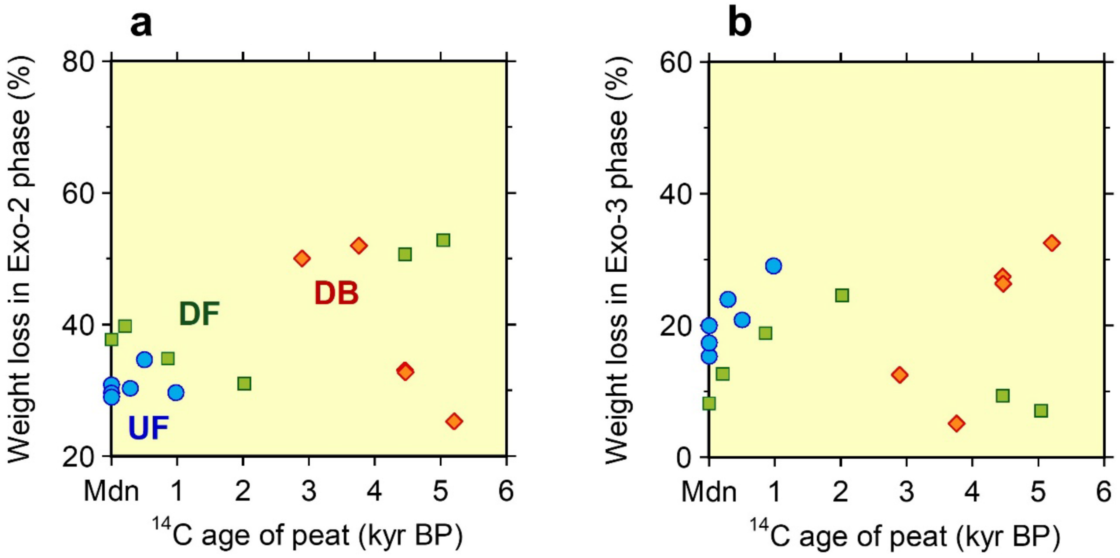


**Supplementary Fig. 4.** Relationships of $^{14}C$ age of peat with thermogravimetry weight losses associated with the exothermal Exo-2 (**a**) and Exo-3 (**b**) phases in TG–DTA for three peatland sites. UF: undrained swamp forest, DF: drained forest, and DB: drained, burnt ex-forest. Mdn on the X-axis denotes the modern $^{14}C$ age.

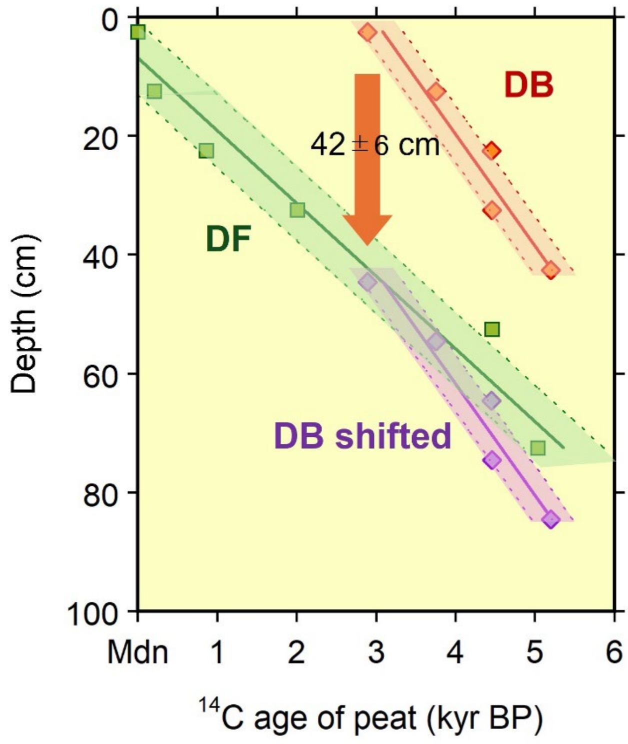


**Supplementary Fig. 5.** Comparison of $^{14}C$ age profiles for the DF (drained forest) and DB (drained, burnt ex-forest) sites. Mdn on the X-axis denotes the modern $^{14}C$ age (set to 0 kyr BP for calculations). The DB profile vertically shifted by 42 cm is also shown. This shift was determined by matching the $^{14}C$ age at a depth of 42 cm in the DF profile ($^{14}C$ age (kyr BP) = −0.561 + 0.082 × depth (cm), $r = 0.98$, $p < 0.001$) with the age (2.89 kyr BP) of the uppermost layer at the DB site.

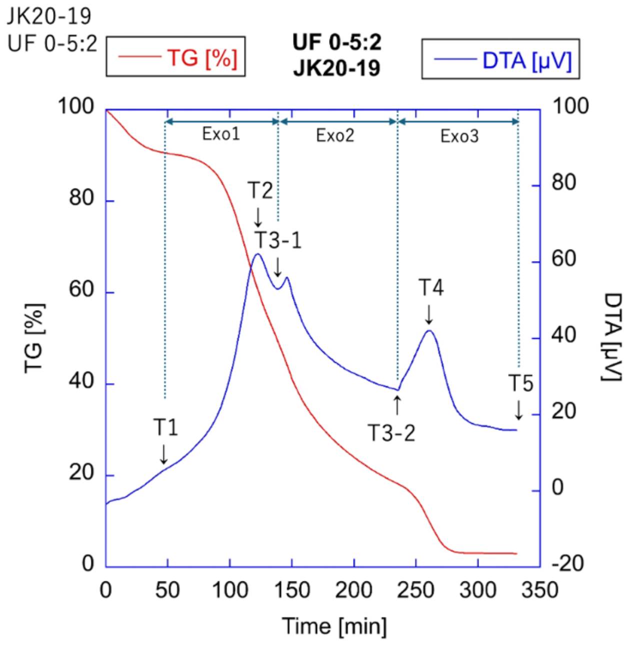


**Supplementary Fig. 6.** TG and DTA curves obtained for a peat sample (UF: 0–5 cm). T1: the onset of the first exothermic peak, T2: the first exothermic peak, T3-1: the minimum point located between the first and second peaks, T3-2: the end of the temperature hold at 375°C, T4: the third exothermic peak.

**Supplementary Table 1.** Carbon stocks and $^{14}C$ ages of peat profiles at three peatland sites

| Site[a] | Depth (cm) | Thickness (cm) | Dry bulk density[b] (g $cm^{-3}$) | C content[b] (%) | C stock (kg C $m^{-2}$) | Cumulative C stock (kg C $m^{-2}$) | $\Delta^{14}C$ (‰) | $^{14}C$ age (yr BP) |
|---|---|---|---|---|---|---|---|---|
| UF | 0–5 | 5 | 0.11 (0.01) | 49.9 (0.2) | 2.7 (0.1) | 2.7 ± 0.1 | 140.8 ± 6.5[c] | Modern |
| | 5–10 | 5 | —[d] | —[d] | *2.7 ± 0.1*[e] | 5.4 ± 0.2 | —[d] | —[c] |
| | 10–15 | 5 | 0.11 (0.00) | 50.3 (0.5) | 2.7 (0.1) | 8.2 ± 0.2 | 70.3 ± 6.2 | Modern |
| | 15–20 | 5 | 0.09 (0.00) | 51.5 (0.5) | 2.3 (0.0) | 10.5 ± 0.2 | 11.5 ± 6.0 | Modern |
| | 20–25 | 5 | 0.09 (0.00) | 51.7 (1.2) | 2.4 (0.1) | 12.9 ± 0.2 | −43.0 ± 5.6 | 290 ± 50[f] |
| | 25–30 | 5 | — | — | *2.5 ± 0.2* | 15.5 ± 0.3 | — | — |
| | 30–35 | 5 | 0.10 (0.01) | 52.6 (0.9) | 2.6 (0.2) | 18.1 ± 0.3 | −67.9 ± 5.6 | 500 ± 50 |
| | 35–40 | 5 | — | — | *2.7 ± 0.2* | 20.8 ± 0.4 | — | — |
| | 40–45 | 5 | 0.10 (0.00) | 54.0 (1.5) | 2.8 (0.1) | 23.6 ± 0.4 | −122.1 ± 5.3 | 980 ± 50 |
| DF | 0–5 | 5 | 0.14 (0.01) | 50.3 (0.3) | 3.5 (0.1) | 3.5 ± 0.1 | 109.6 ± 6.4 | Modern |
| | 5–10 | 5 | — | — | *3.6 ± 0.2* | 7.1 ± 0.2 | — | — |
| | 10–15 | 5 | 0.13 (0.00) | 54.6 (1.4) | 3.6 (0.1) | 10.8 ± 0.3 | −33.6 ± 5.7 | 210 ± 50 |
| | 15–20 | 5 | — | — | *3.2 ± 0.2* | 14.0 ± 0.3 | — | — |
| | 20–25 | 5 | 0.10 (0.00) | 55.0 (0.5) | 2.8 (0.1) | 16.8 ± 0.3 | −108.8 ± 5.1 | 860 ± 50 |
| | 25–30 | 5 | — | — | *4.0 ± 0.2* | 20.8 ± 0.4 | — | — |
| | 30–35 | 5 | 0.16 (0.01) | 63.5 (1.1) | 5.1 (0.2) | 25.9 ± 0.5 | −228.0 ± 4.8 | 2010 ± 50 |
| | 35–50 | 15 | — | — | *13.4 ± 1.1* | 39.3 ± 1.2 | — | — |
| | 50–55 | 5 | 0.14 (0.01) | 53.0 (3.5) | 3.8 (0.3) | 43.0 ± 1.2 | −430.6 ± 3.8 | 4460 ± 50 |
| | 55–70 | 15 | — | — | *10.4 ± 1.0* | 53.4 ± 1.6 | — | — |

| | | | | | | | | |
|---|---|---|---|---|---|---|---|---|
| | 70–75 | 5 | 0.12 (0.01) | 54.8 (1.5) | 3.2 (0.2) | 56.6 ± 1.6 | −470.3 ± 5.1 | 5040 ± 60 |
| DB | 0–5 | 5 | 0.24 (0.01) | 54.4 (1.8) | 6.6 (0.4) | 6.6 ± 0.4 | −307.9 ± 4.5 | 2890 ± 50 |
| | 5–10 | 5 | — | — | *5.2 ± 0.4* | 11.9 ± 0.5 | — | — |
| | 10–15 | 5 | 0.15 (0.01) | 53.5 (1.8) | 3.9 (0.2) | 15.7 ± 0.6 | −378.3 ± 4.1 | 3750 ± 50 |
| | 15–20 | 5 | — | — | *3.7 ± 0.3* | 19.4 ± 0.6 | — | — |
| | 20–25 | 5 | 0.14 (0.01) | 51.8 (2.1) | 3.5 (0.2) | 22.9 ± 0.7 | −430.1 ± 3.8 | 4450 ± 50 |
| | 25–30 | 5 | — | — | *3.7 ± 0.4* | 26.6 ± 0.8 | — | — |
| | 30–35 | 5 | 0.14 (0.01) | 55.1 (4.0) | 3.9 (0.3) | 30.4 ± 0.8 | −430.9 ± 3.8 | 4460 ± 50 |
| | 35–50 | 5 | — | — | *3.4 ± 0.4* | 33.8 ± 0.9 | — | — |
| | 50–55 | 5 | 0.11 (0.00) | 53.4 (2.7) | 2.9 (0.2) | 36.7 ± 0.9 | −480.8 ± 3.7 | 5200 ± 60 |

[a] UF: Undrained swamp forest; DF: Drained forest; DB: Drained, repeatedly burnt ex-forest.

[b] Data are from Itoh et al.[1] Values are mean and standard deviation (in parentheses) of five replicate samples (n = 5).

[c] Errors represent analytical uncertainty (at one standard deviation) in AMS-$^{14}$C measurement.

[d] Uncollected layers.

[e] Values presented in italics represent estimates derived from the mean C stock values of the layers immediately adjacent (above and below) the uncollected layer.

[f] Analytical uncertainty (at one standard deviation) of $^{14}$C dating.

**Supplementary Table 2.** Characteristics of the study sites[2]

| Site[a] | Drainage intensity | Vegetation | Canopy height (m) | Microtopography |
|---|---|---|---|---|
| UF | Little drainage; no large canal, but a network of small canals for illegal logging | *Combretocarpus rotundatus*, *Cratoxylum arborescens*, *Buchanania sessifolia*, and *Tetramerista glabra*, with rich understory shrubbery[4] | 23 | Uneven forest floor with hummocks and hollows |
| DF | Large canal (25 m wide × 3.5–4.5 m deep) excavated in 1996–1997 (ref.[3]) | Same as UF | 26 | Same as UF |
| DB | Same as DF | Ferns (*Stenochlaena, Blechnum* and *Lygodium* spp.) and sedges (*Cyperus*, *Scleria*, and *Eleocharis* spp.) regrown | 0.5 m in June 2005 and 0.8–0.9 m in September 2009 | No hummocks on the ground due to destruction by fires |

[a] UF: Undrained swamp forest; DF: Drained forest; DB: Drained, repeatedly burnt ex-forest.

**Supplementary Table 3.** Thermogravimetry–differential thermal analysis (TG–DTA) parameters determined for peat samples from three peatland sites

| Site[a] | Depth | Temperature in DTA (°C) | | | | | | Remaining weight in TG (%) | | | | | |
|---|---|---|---|---|---|---|---|---|---|---|---|---|---|
| | (cm) | T1 | T2 | T3-1 | T3-2 | T4 | T5 | T1 | T2 | T3-1 | T3-2 | T4 | T5 |
| UF | 0–5 | 57 | 310 | 357 | 380 | 448 | 650 | 93.3 | 61.0 | 49.1 | 18.2 | 9.8 | 2.9 |
| | 10–15 | 53 | 314 | 360 | 380 | 455 | 650 | 94.2 | 60.3 | 48.2 | 18.6 | 8.5 | 1.2 |
| | 15–20 | 49 | 317 | 362 | 381 | 460 | 650 | 96.4 | 63.9 | 53.4 | 24.4 | 12.0 | 4.4 |
| | 20–25 | 50 | 324 | 364 | 381 | 462 | 650 | 96.3 | 65.6 | 56.8 | 26.5 | 10.5 | 2.5 |
| | 30–35 | 52 | 320 | 357 | 381 | 461 | 650 | 95.9 | 67.6 | 59.3 | 24.6 | 11.1 | 3.7 |
| | 40–45 | 52 | 322 | 364 | 381 | 463 | 650 | 95.0 | 68.0 | 58.8 | 29.1 | 10.9 | 0.0 |
| DF | 0–5 | 56 | 310 | 354 | 380 | 432 | 650 | 94.7 | 61.0 | 48.2 | 10.4 | 6.0 | 2.2 |
| | 10–15 | 53 | 315 | 350 | 380 | 444 | 650 | 94.4 | 63.6 | 54.1 | 14.3 | 6.8 | 1.6 |
| | 20–25 | 51 | 317 | 359 | 380 | 449 | 650 | 96.1 | 67.5 | 57.5 | 22.6 | 10.2 | 3.6 |
| | 30–35 | 46 | 317 | 369 | 380 | 447 | 650 | 97.1 | 69.4 | 56.0 | 24.9 | 10.2 | 0.3 |
| | 50–55 | 52 | 319 | 354 | 378 | 430 | 650 | 95.3 | 70.6 | 60.1 | 9.4 | 4.4 | 0.0 |
| | 70–75 | 39 | 313 | 351 | 379 | 423 | 650 | 99.4 | 75.9 | 64.5 | 11.6 | 8.0 | 4.5 |
| DB | 0–5 | 53 | 321 | 346 | 378 | 442 | 650 | 94.3 | 71.5 | 64.9 | 14.8 | 7.6 | 2.1 |
| | 10–15 | 51 | 316 | 352 | 378 | 429 | 650 | 95.6 | 70.4 | 59.3 | 7.3 | 4.5 | 2.1 |
| | 20–25 | 81 | 341 | 369 | 375 | 447 | 650 | 92.5 | 69.1 | 61.9 | 28.8 | 12.8 | 1.3 |
| | 30–35 | 86 | 341 | 372 | 375 | 444 | 650 | 92.4 | 68.9 | 60.8 | 28.1 | 13.5 | 1.6 |
| | 50–55 | 88 | 335 | 376 | 375 | 453 | 650 | 93.7 | 71.7 | 61.0 | 35.7 | 16.0 | 3.2 |

[a] UF: Undrained swamp forest; DF: Drained forest; DB: Drained, repeatedly burnt ex-forest.

**Supplementary Table 4.** Concentrations and $^{14}C$ ages of dissolved organic carbon (DOC) in groundwater at three peatland sites

| Site[a] | Sampling date | GWL[b] (m) | DOC concentration (mg C $L^{-1}$) | $^{14}C$ age (years BP) | Remarks |
|---|---|---|---|---|---|
| UF | December 2013 | 0.00 | 57.3 | Modern | |
| | September 2014 | −0.59 | 68.5 | Modern | |
| | | | | Modern[c] | |
| | December 2015 | −0.11 | 62.6 | Modern | Wet season |
| | June 2016 | −0.07 | 57.7 | Modern | Transition |
| | August 2016 | −0.38 | 68.2 | Modern | Dry season |
| DF | December 2013 | −0.38 | 23.3 | 1660 ± 40[d] | |
| | September 2014 | −1.03 | 81.4 | 730 ± 30 | |
| | December 2015 | −0.34 | 86.3 | 280 ± 60 | Wet season |
| | June 2016 | −0.31 | 76.8 | Modern | Transition |
| | August 2016 | −0.64 | 72.7 | 410 ± 60 | Dry season |
| DB | December 2013 | 0.03 | 21.8 | ND[e] | |
| | September 2014 | −0.60 | 294.6 | 4060 ± 50 | Immediately after a fire event |
| | | | | 3900 ± 40[c] | |
| | December 2015 | −0.33 | 60.8 | 1560 ± 60 | Wet season |
| | June 2016 | −0.36 | 57.2 | 1840 ± 60 | Transition |
| | August 2016 | −0.85 | 95.3 | 2700 ± 60 | Dry season |

[a] UF: Undrained swamp forest; DF: Drained forest; DB: Drained, repeatedly burnt ex-forest.

[b] GWL: Groundwater level. Data from Hirano et al.[5].

[c] The sample used for this $^{14}C$ analysis was identical to the one presented in the preceding row and was prepared as a solid powder by freeze-drying the water sample immediately after collection.

[d] Analytical uncertainty (at one standard deviation) of $^{14}C$ dating.

[e] Not determined due to a failure in the sample preparation process for $^{14}C$ analysis.

**Supplementary Table 5.** Impacts of disturbances (drainage and fires) on peat carbon store in Indonesia

| | |
|---|---|
| *Peat carbon losses estimated in this study* | |
| Peat carbon loss (t C $ha^{-1}$) due to drainage for the period 1996–2014 | 80 (49–111) |
| Peat carbon loss (t C $ha^{-1}$) due to fire events for the period 1996–2014 | 276 (228–325) |
| Total peat carbon loss (t C $ha^{-1}$) due to disturbances for the period 1996–2014 | 356 (298–414) |
| *Extrapolated values for whole of Indonesia* | |
| Peatland area in Indonesia[a] (Mha) | 20.70 |
| Carbon store[a] (Gt C) | 57.37 |
| Area of drained peatland[b] (Mha) | 10.86–13.45 |
| Area of fire-damaged peatland[c] (Mha) | 1.45–6.80 |
| Peat carbon loss (Gt C) due to drainage for the period 1996–2014 | 0.48–1.49 |
| Peat carbon loss (Gt C) due to fire events for the period 1996–2014 | 0.33–2.21 |
| Total peat carbon loss (Gt C) due to disturbances for the period 1996–2014 | 0.81–3.70 |
| Peat carbon loss from store (%) | 1.4–6.4 |

[a] Page et al.[6]

[b] The estimated area of drained peatland in Indonesia was calculated by multiplying the total peatland area (20.70 Mha)[6] by an assumed proportion of drained peatland (65% or 48%). The 65% proportion is an estimate for an area encompassing 157,000 $km^2$ across Sumatra, Borneo, and Peninsular Malaysia[7]. The 48% proportion is based on decadal mean peatland area data for different land cover types from 2011–2020 (ref.[8]). This was calculated by summing the areas of drained peat swamp forest, oil palm plantation, farmland, and drained shrubland, and dividing the sum by the total peatland area in Indonesia (excluding Papua).

[c] Page et al.[9]

**Supplementary Table 6.** Calibrated ages of peat

| Site[a] | Depth (cm) | Calibrated calendar age (cal BP)[b] (2σ) | Median probability (cal BP)[b] |
|---|---|---|---|
| UF | 0–5 | Modern | Modern |
| | 10–15 | Modern | Modern |
| | 15–20 | Modern | Modern |
| | 20–25 | 0–7 (0.8%), 152–171 (4.3%), 179–183 (0.3%), 279–491 (94.6%) | 374 |
| | 30–35 | 472–562 (84.8%), 589–633 (15.0%), 637–639 (0.2%) | 529 |
| | 40–45 | 747– 759 (1.3%), 771–959 (98.7%) | 863 |
| DF | 0–5 | Modern | Modern |
| | 10–15 | 0–45 (15.3%), 57–119 (9.3%), 126–231 (44.1%), 239–318 (28.0%), 394–424 (3.3%) | 187 |
| | 20–25 | 680–832 (82.1%), 844–858 (2.6%), 864–906 (15.2%) | 765 |
| | 30–35 | 1826–2073 (97.3%), 2077–2101 (2.7%) | 1946 |
| | 50–55 | 4883–4931 (5.4%), 4958–5295 (94.6%) | 5118 |
| | 70–75 | 5606–5625 (3.1%), 5652–5911 (96.9%) | 5790 |
| DB | 0–5 | 2877–3166 (100%) | 3026 |
| | 10–15 | 3930–3944 (1.2%), 3969–4251 (97.8%), 4272–4288 (1.0%) | 4111 |
| | 20–25 | 4878–4939 (9.5%), 4955–5149 (51.9%), 5152–5289 (38.6%) | 5101 |
| | 30–35 | 4883–4931 (5.4%), 4958–5295 (94.6%) | 5118 |
| | 50–55 | 5757–5820 (7.0%), 5887–6120 (85.5%), 6141–6182 (7.5%) | 5970 |

[a] UF: Undrained swamp forest; DF: Drained forest; DB: Drained, repeatedly burnt ex-forest.

[b] Calibrated using Calib 8.20 software[10] with the IntCal20 radiocarbon calibration curve[11].

**Supplementary Table 7.** Calibrated ages of dissolved organic carbon (DOC) in groundwater

| Site[a] | Sampling date | Calibrated calendar age (cal BP)[b] (2σ) | Median probability (cal BP)[b] |
|---|---|---|---|
| UF | December 2013 | Modern | Modern |
| | September 2014 | Modern | Modern |
| | | Modern | Modern |
| | December 2015 | Modern | Modern |
| | June 2016 | Modern | Modern |
| | August 2016 | Modern | Modern |
| DF | December 2013 | 1413–1459 (14.1%), 1469–1621 (77.4%), 1669–1692 (8.5%) | 1547 |
| | September 2014 | 572–581 (4.0%), 649–697 (88.7%), 703–723 (7.3%) | 673 |
| | December 2015 | 0–22 (3.1%), 147–191 (9.6%), 193–217 (2.9%), 266–492 (84.4%) | 353 |
| | June 2016 | Modern | Modern |
| | August 2016 | 314–413 (37.5%), 419–529 (62.5%) | 451 |
| DB | December 2013 | Modern | Modern |
| | September 2014 | 4417–4652 (82.0%), 4669–4704 (5.5%), 4754–4810 (12.5%) | 4555 |
| | | 4157–4173 (1.6%), 4177–4291 (3.1%), 4229–4422 (95.3%) | 4331 |
| | December 2015 | 1311–1331 (2.4%), 1341–1545 (97.6%) | 1447 |
| | June 2016 | 1588–1595 (0.4%), 1598–1679 (19.6%), 1684–1890 (79.3%), 1910–1920 (0.7%) | 1748 |
| | August 2016 | 2737–2939 (100%) | 2815 |

[a] UF: Undrained swamp forest; DF: Drained forest; DB: Drained, repeatedly burnt ex-forest.

[b] Calibrated using Calib 8.20 software[10] with the IntCal20 radiocarbon calibration curve[11].

**Supplementary Method 1.** Detailed descriptions of the study sites

The study sites are located in the upper catchment of the Sebangau River, near Palangka Raya, the capital of Central Kalimantan province, Indonesia. This region is characterized by extensive peat domes, with the maximum recorded peat thickness reaching 13 m and an average of 4.4 m to the east of the Sebangau River[9,12]. The study sites are situated relatively close to the edge of these peat domes.

These sites have been the subject of extensive scientific investigation for several decades, employing various methodologies to study diverse ecological aspects[1,2,5,8,9,12-15]. Notably, a substantial area of peatland in Central Kalimantan underwent deforestation and drainage during the late 1990s to establish agricultural land under the national Mega Rice Project (MRP). Although the MRP concluded in 1999, it resulted in widespread, degraded peatland ecosystems.

The three designed sites (UF, DF, and DB) are geographically proximate[13], located within 15 km on relatively flat terrain to the east of the Sebangau River. This close proximity facilitates a comparative assessment of disturbance impacts on C dynamics within peatlands[1,2,5,8,9,12-15]. Detailed site characteristics are documented in Hirano et al.[2]. Key features of each site are summarized in Supplementary Table 2.

The UF site (2.32°S, 113.90°E) represents a largely undisturbed swamp forest, experiencing minimal drainage but subjected to selective logging prior to the late 1990s, resulting in a near-secondary forest condition. The site was in a National Park designated in 2006 and was slightly drained by small ditches made for logging; however, the shallow ditches were mostly buried naturally[5]. While no major canals exist at this site, a network of small canals from past logging activities influences local hydrological conditions[3]. Dominant tree species include *Combretocarpus rotundatus*, *Cratoxylum arborescens*, *Buchanania sessifolia*, and *Tetramerista glabra*, accompanied by a dense understory of shrubs maily composed of their sapling[2,4,16]. The soil surface is characterized by a thick litter layer. Herbaceous vegetation is sparse on the soil surface. The forest floor exhibits topographic variation with hummocks and hollows; hummocks, formed by dense tree root systems, are typically 20–30 cm higher than the surrounding hollows[17]. The peat depth was approximately 3 m (ref.[1]).

The DF site (2.35°S, 114.04°E), formerly an undisturbed swamp forest, is a secondary forest located within Block C of the former MRP area. This forest also experienced selective logging until the late 1990s. Dominant tree species and the microtopography of the forest floor are similar to those at the UF site. A large canal (originally 25 m wide × 3.5–4.5 m deep) was excavated in 1996–1997 as part of the

MRP infrastructure, effectively facilitating drainage of the forest[3]. The peat depth was approximately 4.5 m (ref.[1]).

The DB site (2.34°S, 114.04°E), formerly an undisturbed swamp forest, is located near the DF site. It also experienced selective logging until the late 1990s and has been drained since 1996–1997. This site experienced multiple fires in 1997, 2002, 2009, and 2014, coinciding with El Niño years, which resulted in significant vegetation and surface peat soil losses. A stand-replacing fire in 2002 severely damaged the forest, leaving a few standing dead trees and a considerable amount of coarse woody debris on the ground[15]. The average thickness of burnt peat by the 2002 fire was estimated at 0.22 m (ref.[18]). Data on the combustion thickness caused by the 1997 fire are unavailable. By June 2005, ferns (*Stenochlaena*, *Blechnum*, and *Lygodium* spp.) and sedges (*Cyperus*, *Scleria*, and *Eleocharis* spp.) had grown up to 0.5 m, covering a significant portion of the ground. By September 2009, plant height had increased to 0.8–0.9 m, and some young trees (dominated by *Combretocarpus rotundatus*) had begun to regenerate. A fire in September 2009 caused moderate burning but did not result in stand replacement. Aboveground parts of herbaceous plants and course woody debris on the ground were burned, and the peat surface experienced localized burning[15]. Subsequently, surviving trees initiated leafing under high groundwater level conditions, and ferns and sedges rapidly regrew, covering most of the ground by December 2009. A light-severity fire in September 2014 burned herbaceous plants, accumulated litter on the ground, and young trees in isolated areas, although many trees survived. The fires eliminated any existing pre-existing hummocks. The peat depth was approximately 4.5 m (ref.[1]).

Net ecosystem $CO_2$ exchange measurements have been conducted at all three sites using towers installed in 2001 (DF site) and 2004 (UF and DB sites)[2].

**Supplementary Method 2.** Evaluating C losses due to drainage and fires

Carbon losses due to disturbances were evaluated based on differences in cumulative C stocks (kg C $m^{-2}$) as a function of $^{14}$C age (kyr BP) between the two sites of interest (UF and DF for drainage-induced C loss and DF and DB for fire-induced C loss). For all sites, the cumulative C stocks exhibited significant linear relationships with $^{14}$C age (Fig. 1b; UF: $r = 0.99$, $p < 0.05$, DF: $r = 0.99$, $p < 0.0005$, DB: r = 0,.97; $p < 0.01$) and were estimated with standard errors as follows:

UF: Cumulative C stock = 10.2 + 13.9 × $^{14}$C-age (standard error: 1.1)

DF: Cumulative C stock = 9.2 + 6.9 × $^{14}$C-age (standard error: 3.7)

DB: Cumulative C stock = −32.6 + 13.3 × $^{14}$C-age (standard error: 3.2)

Consequently, the drainage-induced loss of C accumulated over the past 1,000 years was estimated as 8.0 kg C $m^{-2}$ (standard error: 3.1 kg C $m^{-2}$) based on the difference in cumulative C stock between DF and UF (UF minus DF) at a $^{14}$C age of 1 kyr BP (Supplementary Fig. 1). Similarly, the fire-induced loss of C was estimated as 27.6 kg C $m^{-2}$ (standard error: 4.9 kg C $m^{-2}$) based on the difference in cumulative C stock between DF and DB (DF minus DB) at a $^{14}$C age of 2.89 kyr BP, the $^{14}$C age of peat in the uppermost layer of the DB site (Supplementary Table 1).

**Supplementary Method 3.** Evaluating peat depth lost due to fires

Peat depth lost due to fires was evaluated based on the difference in peat depth (cm) as a function of $^{14}C$ age (kyr BP) between the DF and DB sites (Supplementary Fig. 5). For the DF site, the $^{14}C$ age exhibited a significant linear relationship with peat depth (Fig. 1a; DF: $r = 0.98$, $p < 0.001$), and consequently, the peat depth as a function of $^{14}C$ age was estimated with standard error as follows:

DF: Peat depth = ($^{14}C$-age + 0.56) / 0.08 (standard error: 6.2)

The peat depth corresponding to a $^{14}C$ age of 2.89 kyr, the $^{14}C$ age of peat in the uppermost peat layer of the DB site, was then estimated as 42.2 cm (standard error: 6.2 cm).

**Supplementary Method 4.** Estimating weight losses associated with exothermic peaks in TG–DTA

Representative thermogravimetric (TG) and differential thermal analysis (DTA) results for a peat sample (UF: 0–5 cm) are shown in Supplementary Fig. 6 (Results for all samples are summarized in Supplementary Fig. 3). The DTA curve displays three exothermic peaks, although the profile of the second peak was interrupted by a 90-minute temperature hold at 375°C. On the basis of these observed peaks, we defined temperature ranges corresponding to three exothermic reaction phases (Exo-1, Exo-2, and Exo-3). The temperature ranges are listed in Supplementary Table 3.

Exo-1 encompasses the temperature range from the onset of the first exothermic reaction (T1) to the minimum point (T3-1) of the DTA curve located between the first (T2) and second (T3-2) exothermic peaks. Exo-2 encompasses the second peak (T3-2), spanning from T3-1 to the end of the 90-minute temperature hold (T3-2). Exo-3 encompasses the third exothermic peak (T4), extending from T3-2 to 650°C (T5). Subsequently, weight losses associated with each exothermic phase (Exo-1, Exo-2, and Exo-3) were determined from the TG curve as the difference in weigh between the initial and final temperatures defining each phase.

**Supplementary References**


1. Itoh, M., Okimoto, Y., Hirano, T. & Kusin, K. Factors affecting oxidative peat decomposition due to land use in tropical peat swamp forests in Indonesia. *Sci. Total Environ.* **609**, 906-915 (2017).
2. Hirano, T. et al. Effects of disturbances on the carbon balance of tropical peat swamp forests. *Glob. Change Biol.* **18**, 3410-3422 (2012).
3. Page et al. Restoration ecology of lowland tropical peatlands in Southeast Asia: Current knowledge and future research directions. *Ecosystems* **12**, 888-905 (2009).
4. Tuah, S. J., Jamal, Y. M. & Limin, S. H. Nutritional characteristics in leaves of plants native to tropical peat swamps and heath forests of Central Kalimantan, Indonesia. *TROPICS* **12**, 221-245 (2003).
5. Hirano, T. et al. Large variation in carbon dioxide emissions from tropical peat swamp forests due to disturbances. *Commun. Earth Environ.* **5**, 221 (2024).
6. Page, S. E., Rieley, J. O. & Banks, C. J. Global and regional importance of the tropical peatland carbon pool. *Glob. Change Biol.* **17**, 798-818 (2011).
7. Dadap, N. C. et al. Drainage canals in Southeast Asian peatlands increase carbon emissions. *AGU Advances* **2**, e2020AV000321 (2021).
8. Hirano, T. et al. Impact of land use change and drought on the net emissions of carbon dioxide and methane from tropical peatlands in Southeast Asia. *AGU Advances* **6**, e2025AV001861 (2025).
9. Page, S. E. et al. The amount of carbon released from peat and forest fires in Indonesia during 1997. *Nature* **420**, 61-65 (2002).
10. Stuiver, M. & Reimer, P. J. Extended $^{14}$C data base and revised CALIB 3.0 $^{14}$C age calibration program. *Radiocarbon* **35**, 215-230 (1993).
11. Reimer, P. J. et al. The IntCal20 Northern Hemisphere radiocarbon age calibration curve (0–55 cal kBP). *Radiocarbon* **62**, 725-757 (2020).
12. Page, S. E. et al. A record of Late Pleistocene and Holocene carbon accumulation and climate change from an equatorial peat bog (Kalimantan, Indonesia): implications for past, present and future carbon dynamics. *J. Quat. Sci.* **19**, 625-635 (2004).
13. Moore, S. et al. Deep instability of deforested tropical peatlands revealed by fluvial organic carbon fluxes. Nature **493**, 660-663 (2013).
14. Könönen, M. et al. Deforested and drained tropical peatland sites show poorer peat substrate quality and lower microbial biomass and activity than unmanaged swamp forest. *Soil Biol. Biochem.* **123**, 229-241 (2018).

15. Ohkubo, S., Hirano, T. & Kusin, K. Assessing the carbon dioxide balance of a degraded tropical peat swamp forest following multiple fire events of different intensities. *Agric. For. Meteorol.* **306**, 108448 (2021).
16. Poesie, E. S. et al. Species composition and phylogenetic diversity in a tropical peat swamp forest, Central Kalimantan, Indonesia. *TROPICS* **19**, 93-105 (2011).
17. Jauhiainen, J., Takahashi, H., Heikkinen, J. E. P., Martikainen, P. J. & Vasander, H. Carbon fluxes from a tropical peat swamp forest floor. *Glob. Change Biol.* **11**, 1788-1797 (2005).
18. Hirano, T., Kusin, K., Limin, S. & Osaki, M. Carbon dioxide emissions through oxidative peat decomposition on a burnt tropical peatland. *Glob. Change Biol.* **20**, 555-565 (2014).
