# Supplementary material for: Progressive release of long-stored carbon from tropical peatland disturbances
Source: Nat Commun. 2026 May 27;17:4369. doi: 10.1038/s41467-026-72890-y (PMC13216611; doi:10.1038/s41467-026-72890-y)
Supplement: Supplementary file 2 — Reporting Summary [file 41467_2026_72890_MOESM2_ESM.pdf]

nature portfolio

Corresponding author(s): Jun Koarashi

Last updated by author(s): Apr 8, 2026

Reporting Summary

Nature Portfolio wishes to improve the reproducibility of the work that we publish. This form provides structure for consistency and transparency in reporting. For further information on Nature Portfolio policies, see our Editorial Policies and the Editorial Policy Checklist.

Statistics

For all statistical analyses, confirm that the following items are present in the figure legend, table legend, main text, or Methods section.

n/a	Confirmed	
☐	☒	The exact sample size (*n*) for each experimental group/condition, given as a discrete number and unit of measurement
☐	☒	A statement on whether measurements were taken from distinct samples or whether the same sample was measured repeatedly
☒	☐	The statistical test(s) used AND whether they are one- or two-sided<br>*Only common tests should be described solely by name; describe more complex techniques in the Methods section.*
☒	☐	A description of all covariates tested
☒	☐	A description of any assumptions or corrections, such as tests of normality and adjustment for multiple comparisons
☒	☐	A full description of the statistical parameters including central tendency (e.g. means) or other basic estimates (e.g. regression coefficient) AND variation (e.g. standard deviation) or associated estimates of uncertainty (e.g. confidence intervals)
☒	☐	For null hypothesis testing, the test statistic (e.g. *F*, *t*, *r*) with confidence intervals, effect sizes, degrees of freedom and *P* value noted<br>*Give P values as exact values whenever suitable.*
☒	☐	For Bayesian analysis, information on the choice of priors and Markov chain Monte Carlo settings
☒	☐	For hierarchical and complex designs, identification of the appropriate level for tests and full reporting of outcomes
☒	☐	Estimates of effect sizes (e.g. Cohen's *d*, Pearson's *r*), indicating how they were calculated

Our web collection on statistics for biologists contains articles on many of the points above.

Software and code

Policy information about availability of computer code

Data collection	Radiocarbon analysis was performed using JAEA-AMS-TONO-5MV (a 5 MV tandem Pelletron type accelerator: Model 15SDH-2, National Electrostatics Corporation, USA) and JAEA-AMS-MUTSU (a 3MV Tandetron system: Model 4130, High Voltage Engineering Europe).<br>TG-DTA analysis was performed with Thermo plus EVO2 TG-DTA8122 (Rigaku, Japan).<br>Elemental analysis was performed with a CN analyser (Sumigraph NCH-22, Sumika Chemical Analysis Service Ltd., Japan) for peat and a TOC-V CPH (Shimadzu, Japan) for groundwater dissolved organic matter.
Data analysis	No custom code or software was used for the analysis and presentation of the data associated with this article.<br>Radiocarbon analysis was performed using NEC AMS Analysis Program “abc” Ver.6.1 (JAEA-AMS-TONO) and Dating ver16.00, Nov.2012 and Source ver6.00, May 2009 (JAEA-AMS-MUTSU).<br>Calibrated ages were calculated using Calib 8.20 software with the IntCal20 radiocarbon calibration curve.<br>TG-DTA analysis was performed with Thermo plus EVO2 Version 2.5.3.0-2 (Rigaku, Japan) and QuadVision Version 3.38 for Windows (Rigaku, Japan).

For manuscripts utilizing custom algorithms or software that are central to the research but not yet described in published literature, software must be made available to editors and reviewers. We strongly encourage code deposition in a community repository (e.g. GitHub). See the Nature Portfolio guidelines for submitting code & software for further information.

Data

Policy information about availability of data

All manuscripts must include a data availability statement. This statement should provide the following information, where applicable:

- Accession codes, unique identifiers, or web links for publicly available datasets
- A description of any restrictions on data availability
- For clinical datasets or third party data, please ensure that the statement adheres to our policy

The data generated in this study are provided in the Supplementary Information file.

Research involving human participants, their data, or biological material

Policy information about studies with human participants or human data. See also policy information about sex, gender (identity/presentation), and sexual orientation and race, ethnicity and racism.

Reporting on sex and gender	n/a
Reporting on race, ethnicity, or other socially relevant groupings	n/a
Population characteristics	n/a
Recruitment	n/a
Ethics oversight	n/a

Note that full information on the approval of the study protocol must also be provided in the manuscript.

Field-specific reporting

Please select the one below that is the best fit for your research. If you are not sure, read the appropriate sections before making your selection.

☐ Life sciences ☐ Behavioural & social sciences ☒ Ecological, evolutionary & environmental sciences

For a reference copy of the document with all sections, see nature.com/documents/nr-reporting-summary-flat.pdf

Ecological, evolutionary & environmental sciences study design

All studies must disclose on these points even when the disclosure is negative.

Study description	Peat and groundwater sampling sites were selected from well-established areas that have previously been studied by using various monitoring methods, enabling result validation. Sites were chosen across a disturbance sequence to assess the impact of sequential disturbances on carbon storage and release in a representative tropical peat landscape. These sites have similar topographies, vegetation types, and peat thicknesses.
Research sample	Peat samples were collected from a face of pit at each site. Groundwater samples were collected near the groundwater surface using installed wells equipped with strainers.
Sampling strategy	Peat samples were collected via the pit excavation method, providing high depth-resolution from well-preserved sites, rather than from the peat surface using standard core sampling. This approach was specifically chosen to prevent contamination of deeper peat samples (which are largely depleted in 14C due to radioactive decay) by shallower samples (rich in modern C and high in 14C). Contamination during core sampling is a common issue that can significantly affect 14C age determination. However, the pit excavation method we used restricted the depth of peat samples we could collect. We made every effort to conduct peat sampling in September 2014, considering the generally lowest groundwater levels of the year and the unusually low groundwater levels associated with the 2014 El Niño drought. Deeper peat layers were almost permanently flooded at all sites and were considered well-preserved, with minimal influence from drainage or fires. A limitation is the lack of replicated peat profile samples, which restricts assessment of heterogeneity and uncertainty, particularly at the fire-affected site. This limitation is described in the manuscript.
Data collection	The collected data include radiocarbon and carbon content of peat, as well as dissolved organic matter in groundwater, and thermal decomposition properties for peat.
Timing and spatial scale	Peat samples were collected in 2014, 18 years after drainage by canal excavation commenced at drained sites. The fire-affected site experienced repeated fires in 1997, 2002, 2009, and 2014 during this 18-year period, enabling assessment of fire's impact on peat carbon dynamics. Groundwater samples were collected five times between 2013 and 2016 to identify inter-annual patterns. These sites are located within 15 km of tropical peatlands within an edaphycally similar area.

Data exclusions	No data were excluded from the study.
Reproducibility	Radiocarbon analysis was conducted using established instruments and methods, yielding high accuracy (with an analytical uncertainty of less than 60 years for conventional radiocarbon age dating).
Randomization	The study was rondomised to the extent that study sites were selected solely based on the availability of appropriate, undisturbed (excluding disturbances specifically related to this study) locations. Within each site, samples were non-rondomly collected at a representative location, excluding tree gaps, uneven surface, and flooded areas.
Blinding	Blinding was not relevant to this study.

Did the study involve field work? ☒ Yes ☐ No

Field work, collection and transport

Field conditions	Peat samples were collected in September because of the lowest groundwater levels in the peat profile, enabling deeper sampling. Groundwater samples were collected across different years and seasons to assess changes in dissolved organic carbon ages since the disturbances and related to groundwater level fluctuations.
Location	Latitude, longitude, and mean annual groundwater level of our study sites in peatland, an undrained swamp forest (UF), a drained forest (DF), and a drained, repeatedly burnt ex-forest (DB) in Palangkaraya, Central Kalimantan, Indonesia, are listed in Table 1.
Access & import/export	The work in the peatland sites was carried out under research permits obtained from the Indonesian Ministry for Research and Technology, and the export and import of the specimens were conducted with the appropriate agreements between the relevant authorities.
Disturbance	Soil pits were excavated to a depth of approximately 80 cm and with an area of less than 1 m x 1 m for peat sample collection. To minimize environmental impact, the pits were backfilled after sampling.

Reporting for specific materials, systems and methods

We require information from authors about some types of materials, experimental systems and methods used in many studies. Here, indicate whether each material, system or method listed is relevant to your study. If you are not sure if a list item applies to your research, read the appropriate section before selecting a response.

Materials & experimental systems

n/a	Involved in the study	
☒	☐	Antibodies
☒	☐	Eukaryotic cell lines
☒	☐	Palaeontology and archaeology
☒	☐	Animals and other organisms
☒	☐	Clinical data
☒	☐	Dual use research of concern
☒	☐	Plants

Methods

n/a	Involved in the study	
☒	☐	ChIP-seq
☒	☐	Flow cytometry
☒	☐	MRI-based neuroimaging

Plants

Seed stocks	n/a
Novel plant genotypes	n/a
Authentication	n/a
